# Temporal Cortex Activation to Audiovisual Speech in Normal-Hearing and Cochlear Implant Users Measured with Functional Near-Infrared Spectroscopy

**DOI:** 10.3389/fnhum.2016.00048

**Published:** 2016-02-11

**Authors:** Luuk P. H. van de Rijt, A. John van Opstal, Emmanuel A. M. Mylanus, Louise V. Straatman, Hai Yin Hu, Ad F. M. Snik, Marc M. van Wanrooij

**Affiliations:** ^1^Department of Otorhinolaryngology, Donders Institute for Brain, Cognition, and Behaviour, Radboud University Nijmegen Medical CentreNijmegen, Netherlands; ^2^Department of Biophysics, Donders Institute for Brain, Cognition, and Behaviour, Radboud University NijmegenNijmegen, Netherlands

**Keywords:** functional near-infrared spectroscopy (fNIRS), audiovisual, auditory cortex, cochlear implants, speech

## Abstract

**Background**: Speech understanding may rely not only on auditory, but also on visual information. Non-invasive functional neuroimaging techniques can expose the neural processes underlying the integration of multisensory processes required for speech understanding in humans. Nevertheless, noise (from functional MRI, fMRI) limits the usefulness in auditory experiments, and electromagnetic artifacts caused by electronic implants worn by subjects can severely distort the scans (EEG, fMRI). Therefore, we assessed audio-visual activation of temporal cortex with a silent, optical neuroimaging technique: functional near-infrared spectroscopy (fNIRS).

**Methods**: We studied temporal cortical activation as represented by concentration changes of oxy- and deoxy-hemoglobin in four, easy-to-apply fNIRS optical channels of 33 normal-hearing adult subjects and five post-lingually deaf cochlear implant (CI) users in response to supra-threshold unisensory auditory and visual, as well as to congruent auditory-visual speech stimuli.

**Results**: Activation effects were not visible from single fNIRS channels. However, by discounting physiological noise through reference channel subtraction (RCS), auditory, visual and audiovisual (AV) speech stimuli evoked concentration changes for all sensory modalities in both cohorts (*p* < 0.001). Auditory stimulation evoked larger concentration changes than visual stimuli (*p* < 0.001). A saturation effect was observed for the AV condition.

**Conclusions**: Physiological, systemic noise can be removed from fNIRS signals by RCS. The observed multisensory enhancement of an auditory cortical channel can be plausibly described by a simple addition of the auditory and visual signals with saturation.

## Introduction

Viewing a talking person’s face and mouth may enhance speech understanding in noisy environments (MacLeod and Summerfield, 1990; Helfer, 1997). This effect is due to multisensory integration, in which congruent unisensory signals from multiple modalities are merged to form a coherent and enhanced percept (Stein and Meredith, 1993). The mechanisms underlying multisensory integration have been studied extensively at the single-neuron level in animals (review on seminal work in anesthetized cat; Stein and Meredith, 1993), and in psychophysical eye movement studies in humans (Corneil et al., 2002; Van Barneveld and Van Wanrooij, 2013). How these mechanisms relate to the neural underpinnings of human speech recognition has been studied with neuroimaging and electrophysiological techniques (Calvert et al., 2004; Beauchamp, 2005; Stein, 2012). In individual neurons, the multisensory responses can be much greater than the linear sum of individual unisensory responses. In contrast, for fMRI data, integrating across millions of neurons, super-additivity is typically not found, although multisensory responses are slightly greater than the maximum or mean of the individual unisensory responses (Laurienti et al., 2005).

Here, we attempt to characterize multisensory speech processing by applying an alternative, non-invasive method to record neural activity: functional near-infrared spectroscopy (fNIRS). fNIRS assesses cortical hemodynamic changes in blood oxygenation based on changes in the transmission of near-infrared light through biological tissue and its absorption by oxygenated (HbO_2_) and deoxygenated (HbR) hemoglobin (Jöbsis, 1977; Cope and Delpy, 1988; Huppert et al., 2006, 2009a; Abdelnour and Huppert, 2009). As fNIRS is a non-invasive, minimally-restrictive and quiet optical technique [as opposed to PET (Johnsrude et al., 2002) and fMRI (Hall et al., 2000)], it is ideally suited for auditory studies (Plichta et al., 2011; Pollonini et al., 2014; Santosa et al., 2014; Chen et al., 2015b) on human subjects of all ages. Furthermore, this technique does not suffer from the severe limitations imposed by electro-magnetic implants (e.g., cochlear implant, CI; Gilley et al., 2006). Therefore, it has been successfully used to study human auditory cortex activation by speech stimuli in normal-hearing adults (Pollonini et al., 2014) and deaf adults and children using a CI (Sevy et al., 2010; Chen et al., 2015a; Dewey and Hartley, 2015).

**Figure 1:**
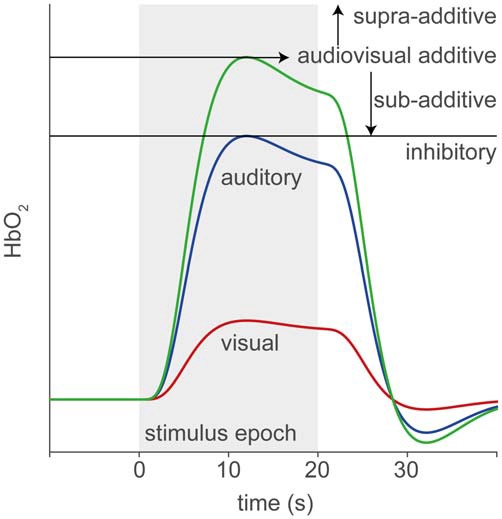
**Rationale of audiovisual (AV) fNIRS experiments.** Hemodynamic responses taken from temporal cortex will differ for the different stimulus modalities, such that to an auditory (blue line) stimulus (gray patch) the response amplitude is larger than to a visual (red) stimulus. We test for potential multisensory integration at the level of temporal cortex, by comparing the hemodynamic response to bimodal stimulus presentation (green) to the linear sum of the visual and auditory responses (additive). Supra- or sub-additive effects on the AV response may be a signature for AV integration.

In this study, we use fNIRS to record supra-threshold auditory, visual and audiovisual (AV) speech-evoked activity from temporal cortex of normal-hearing adults and post-lingually deaf unilateral CI users. We use a limited number of fNIRS channels in order to reduce the time and complexity of applying the optodes. Figure 1 illustrates the rationale and possible outcomes of our experiments. Pure auditory stimulation is expected to produce a typical hemodynamic response profile (blue, Smith, 2004; Malinen et al., 2007) in line with the BOLD response (for review, see Steinbrink et al., 2006; Cui et al., 2011), that reaches its peak at about 6–10 s after a transient stimulus onset. In contrast, pure visual stimulation may produce at best a lower response (red) for a predominantly auditory-responsive area, which could be due to the expectation of a sound being produced by the moving lips (Calvert et al., 1997). Evidence for clear audiovisual integration would be found if the AV response exceeds mere linear summation of the two unimodal responses, i.e., the additive response, or when it falls below the unisensory auditory response (inhibition; Stein et al., 2009). A sub-additive response might be due to either a multisensory or nonlinear saturation effect.

We also tested a limited number of post-lingually deaf unilateral CI users mainly to examine the feasibility of recording multisensory speech processing at the level of temporal cortex with easily-applied, 4-channel fNIRS in the presence of electrical innervation of the auditory nerve by a CI.

## Materials and Methods

### Subjects

Thirty-three adult native Dutch-speaking normal-hearing subjects (age: 18–62 years, median 29, 15 male, 18 female) and five adult Dutch-speaking post-lingually deaf unilateral CI users (age: 55–59 years, median 57, all female, Table 1) were recruited to participate in this study. All normal-hearing subjects (within 20 dB of audiometric zero, range 0.5–8 kHz) and all CI users had normal or corrected to normal vision. Experiments were conducted after obtaining written consent from the subject. The experiments were approved by the Ethics Committee of Arnhem-Nijmegen (project number NL24364.091.08, October 18, 2011) and were carried out in accordance with the relevant institutional and national regulations and with the World Medical Association Helsinki Declaration as revised in October 2008^1^.

**Table 1:** **Subject demographics of post-lingually deaf cochlear implant (CI) users**.

CI user	Implanted ear	Etiology	Cochlear implant use (years)	Device
P1	Left	Cogan syndrome	12	C2HighFocus2^1^
P2	Right	Progressive	5	Nucleus24RCA^2^
P3	Left	Progressive	8	C1^1^
P4	Left	Sudden deafness	19	Nucleus 22^2^
P5	Left	Progressive	7	Nucleus24RCS^2^

*^1^Advanced Bionics, Stäfa, Switzerland. ^2^Cochlear Headquarters, Sydney, Australia*.

### Experimental Setup

Subjects sat comfortably in a reclining chair, to reduce head movements and to minimize low-frequency so-called Mayer waves, that are presumably caused by slow variations in blood pressure (Julien, 2006). The experiment was performed in a darkened experimental room (3.2 × 3.2 × 3.5 m) in which the walls and the ceiling were covered with black acoustic foam that eliminated echoes for sound frequencies >500 Hz (Agterberg et al., 2011). Background noise level was less than 30 dB, A-weighted (dBA; Bremen et al., 2010).

fNIRS data were collected with a pulsed continuous-wave NIRS instrument with four optical sources and two photodetectors (Oxymon MKIII Near-Infrared Spectrophotometer, Artinis Medical Systems BV, Elst, Netherlands; for a comprehensive review of the principles and practicalities of continuous-wave fNIRS, see e.g., Scholkmann et al., 2014). Each optical source consisted of two lasers with emission wavelengths of 858 or 861 nm and 765 nm.

**Figure 2:**
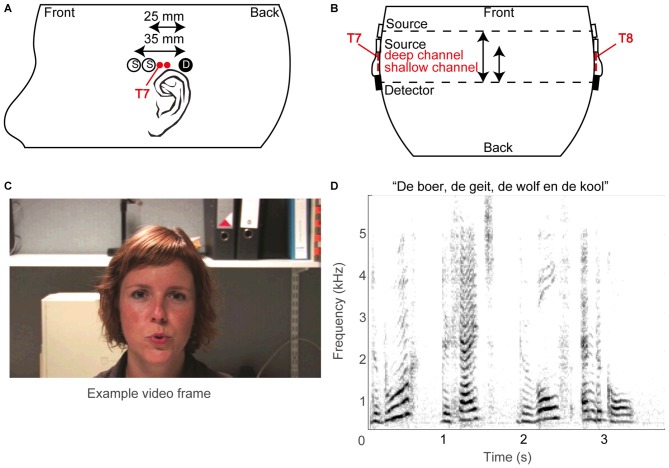
**Methodological overview. (A)** Schematic layout of optical sources (open circles) and photo detectors (filled circles) on the left hemisphere, and **(B)** schematic top view of probe layout. The estimated T7 and T8 positions of the 10/20 system are also indicated, as are the supposed superficial centers of the deep and shallow channels (red filled circles). **(C)** An example video frame. **(D)** A spectrogram of an example sound snippet (the title shows the first words of the story).

The fNIRS probe template (Figures 2A,B) consisted of two optical sources and a single detector, typically on both sides of the head (see below), with source-detector distances of 25 and 35 mm, termed reference or shallow and deep channel, respectively. Sources and detectors were embedded in plastic molds, which were secured in place on the skull by adjustable straps. The temporal cortex was located based on the 10–20 system (Jasper, 1958), which roughly estimates its location at T7 for the left hemisphere and T8 for the right hemisphere (Herwig et al., 2003; Figures 2A,B). As fNIRS measures brain activity over a diffuse area, we did not pinpoint the exact cortical areas per subject: based on Monte Carlo simulations by others (Fukui et al., 2003; Haeussinger et al., 2011; Strangman et al., 2014; Brigadoi and Cooper, 2015), the average photon path from source to photodetector is estimated to be an ellipsoid with a penetration depth of approximately 2–3 cm. Specifically, the current fNIRS probe template is expected to cover a large area of the temporal cortex (c.f. Sevy et al., 2010).

For 21 normal-hearing subjects the optodes were positioned by aligning the mid-point of the long-distance (35 mm) source-detector pairs above the preauricular point at the T7 and T8 location of the International 10/20 system on the left and right hemisphere, respectively (Niedermeyer and Lopes da Silva, 2005). For the other 12 normal-hearing subjects, who were measured prior to the other subjects, only one side was recorded (left hemisphere, T7). For the CI users, only the hemisphere contralateral to the implant was measured with two sources and one detector, to avoid placement problems of the optodes over the implant. The straps were adjusted to guarantee secure coupling between optodes and scalp at acceptable comfort levels of the subject. Secure coupling was verified online by the presence of a detectable photon count and of a clear cardiac oscillatory response in the raw NIRS trace measured before the experiment. The optodes were connected via optical fibers to the NIRS instrument. The company’s software Oxysoft controlled data acquisition, and allowed for online observation of the data. The data were stored at a sampling rate of either 10 (for the early measurements, which included 12 normal-hearing subjects and all 5 CI users) or 250 Hz (for later measurements on 21 normal-hearing subjects). For data analysis, the latter data were downsampled to 10 Hz.

### Stimuli

The stimuli were composed of digital video recordings of a female speaker reading aloud children’s stories in Dutch (Figures 2C,D). In the auditory-only condition, the voice was presented without visual input (Figure 2D). In the visual-only condition, the video of the woman reading the story was presented on the screen without the auditory signal (Figure 2C). In the auditory-visual condition, the video was presented with the corresponding auditory input. The recordings were digitally edited into 36 20.5 s segments, each consisting of a single vignette from one of three stories (in Dutch: “De boer, de geit, de wolf en de kool”, “De professor”, and “De prinses”). The three stimulus conditions were presented interleaved in pseudorandom order within a single block. Stimulus generation was controlled by a Dell PC (Dell Inc., Round Rock, TX, USA) running Matlab version 2009b (The Mathworks, Natick, MA, USA) using Psychophysics Toolbox 3 extensions (Brainard, 1997; Pelli, 1997; Kleiner et al., 2007). Sounds were presented through headphones (Sennheiser PCX 350 NoiseGuard, Sennheiser electronic GmbH & CO KG, Wedemark, Lower Saxony, Germany, noise cancellation off) at a comfortable listening volume of 55 dBA, while the video was presented on the Dell PC’s monitor. As the implant interfered with placement of headphones for three out of five CI users, the acoustic stimuli to these CI users was alternatively presented via the direct input to the CI or via a free-field speaker.

### Paradigm

The 36 segments were played in chronological order, each followed by a silent, dark period ranging from 25 to 50 s (randomly drawn from a uniform distribution). Even the shortest intermittent period of 25 s allowed the hemodynamic response to return to baseline, while the randomization limited time locking of any periodic physiological signal to stimulus onsets. The segments were presented in three blocks of 12 stimuli each. A single session consisted of these three blocks with intermittent breaks of about 4–5 min wherein the light was turned on. Every block started with a baseline measurement (in silence and darkness) of 2 min. A session of three blocks (36 segments) took about 45 min to complete.

For every block, the 12 segments were pseudo-randomly assigned to an experimental condition (four segments auditory-only, four segments visual-only, four segments auditory-visual). Subjects were instructed to pay attention to the segments (watching, listening, both), and were asked afterwards whether they understood the gist of the storyline. Other than that, subjects were not given further task instructions.

### Analysis

#### Signal Processing

The optical densities for each channel and wavelength were stored on disk (in the native oxy-format from the Artinis system) for offline analysis in Matlab (Release 2014b, the Mathworks, Inc., Natick, MA, USA). Data was read into Matlab via Artinis’ proprietary function *oxysoft2matlab*. The 250-Hz sample-rate data were downsampled to 10 Hz (using the *resample* function from Matlab’s Signal Processing Toolbox), for computational efficiency.

**Figure 3:**
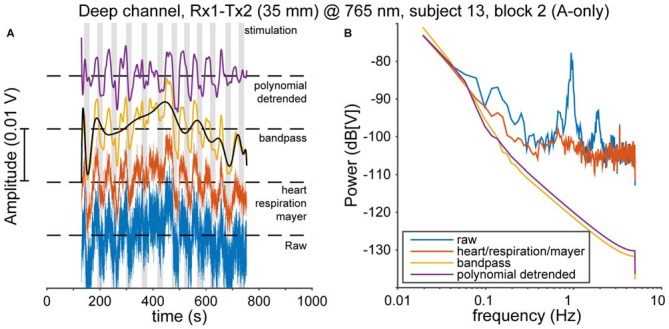
**Pre-processing. (A)** The data are preprocessed in several steps. First, cardiac, respiratory and Mayer oscillations in the raw data (blue, bottom) are removed (red). Then the data are bandpass-filtered between 0.008 and 0.1 Hz (yellow). Subsequently, slow-moving drifts are identified by a polynomial fit (black), which is removed to yield the final signal (purple). **(B)** The effects of every pre-processing step on the power spectrum of the data in **(A)**.

Physiological noise, such as heart pulsation, respiration, and Mayer waves (Huppert et al., 2009b) is mixed with cortical activity in the fNIRS signal. A clear cardiac oscillation is regarded as evidence for a proper contact between the optical probes and the scalp (Themelis et al., 2004). Therefore, following Pollonini et al. (2014), we determined the scalp coupling index (SCI) as the correlation between the two photodetected signals at 765 and ~860 nm, band-pass filtered between 0.5 and 2.5 Hz (typical frequency range for heart rate that excludes low-frequency fNIRS activity), for every optode. Typically, the SCI was highly positive (median 0.98), as expected from physiological signals that have no origin in the neural source (Yamada et al., 2012), and only 24 out of 354 channels [(21 normal-hearing subjects × 2 hemispheres + 12 normal-hearing subjects × 1 hemisphere + 5 CI users × 1 hemisphere) × 2 channels × 3 recording blocks] had an SCI less than 0.9. These 24 low-SCI channels were rejected from further analysis as we deemed those indicative for poor contact between optode and scalp. Then, to remove cardiac, respiratory, and Mayer wave noise sources, we used the *removeheartbeat* function from the NIRS Analysis Package (Fekete et al., 2011); in short, this algorithm extracts an oscillatory template from a narrow-frequency filtered average of all channels per subject (with the filter band containing the oscillatory frequencies of interest), and subtracts this from each channel. Then, we band-pass filtered the signals between 0.008 and 0.1 Hz (Figures 3A,B, red and yellow curves). Next, the data was de-trended using a 20th-degree polynomial in order to remove slow temporal drifts (Figures 3A,B, black and purple curves; Pei et al., 2007). These processed optical densities were converted to changes in oxygenated and deoxygenated hemoglobin concentration (HbO_2_ and HbR, respectively) using the modified Lambert-Beer law (Cope and Delpy, 1988; Kocsis et al., 2006). Subsequently, the preprocessed data were normalized by the variance in each recorded signal for the entire session.

**Figure 4:**
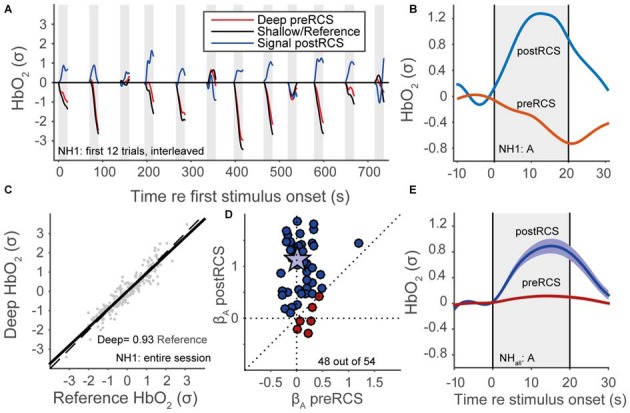
**Reference channel subtraction (RCS). (A)** Normalized HbO_2_ data for a normal-hearing subject (NH1), 12 auditory trials, colors denote deep pre-RCS channel (red), shallow/reference channel (black) and post-RCS/residual signal (blue). **(B)** Averaged normalized HbO_2_ data for 12 auditory stimuli of a normal-hearing subject (NH1). Red line represents data before RCS. The blue line represents data after RCS. **(C)** Linear regression between the deep and the shallow channel HbO_2_ signals. **(D)** Regression coefficients after RCS vs. before RCS (see Statistics); blue indicates improvement, red inhibition, star subject NH1. **(E)** Normal-hearing cohort pre-RCS (red line) and post-RCS (blue line) averages (thick line) and standard error of the means (patch) during auditory stimulation.

Despite these filtering procedures, a considerable amount of noise originating from non-cortical physiological processes still remained (Scholkmann et al., 2014). To deal with this, we applied *reference channel subtraction* (RCS; Scarpa et al., 2013; Brigadoi and Cooper, 2015). This assumes that the shallow channel (the signal originating from the shorter-distance optode source-detector) is dominated by non-cortical signals, while the deep channel (the signal arising from the longer-distance optode source-detector) also includes more of the cortical event-related signal of interest. Therefore, we determined the fNIRS signal as the residual signal from a simple linear regression between the deep and shallow channels (Figure 4C). Note that we applied the normalization of data in the graphs both before and after RCS, so that the signals are scaled with respect to the data variance, and are hence dimensionless. An individual trace of HbO_2_ for a single normal-hearing subject (NH1) for the shallow (black line), deep channel (red line) and the residual signal, during presentation of auditory snippets, is plotted in Figure 4A. An example of how RCS can affect the average evoked response at the single subject-level is shown in Figure 4B. Even though we can expect that the shallow channel might contain some cortical signal because of the relatively large distance of 25 mm, the RCS procedure in general improved the beta coefficients (Figure 4D; see also “GLM” Section) and the signal response (Figure 4E). Because the same (systemic, not event related) noise is present in deep and reference channels, it is successfully removed by RCS. As a result, the variance in the deep channel signal decreases, and hence the signal-to-noise ratio increases. Normalization with a smaller variance leads to an increase of the beta coefficient (Figure 4D), and to the appearance of a clear average activation signal (Figure 4E).

### Statistics

#### Average

Functional signals were averaged across the 12 repeats of each stimulus modality to calculate the average hemodynamic response for each participant and hemisphere. These traces were averaged across participants and hemispheres (no significant hemispheric differences were observed for the bilaterally-measured 21 normal-hearing subjects according to a Wilcoxon signed-rank test, *p* > 0.05 for both HbO_2_ and HbR) to determine the mean response for both cohorts.

#### GLM

We compared both the measured concentration changes of HbO_2_ and HbR to a predicted hemodynamic response function (HRF). The HRF consists of a canonical impulse response function h (as used by the SPM toolbox; Henson and Friston, 2007; Lindquist et al., 2009):

(1) $$h(\tau )=\frac{1}{\Gamma (6)}\tau^{5}e^{-\tau }-\frac{1}{6\Gamma (16)}\tau^{15}e^{-\tau }$$

(with *τ* time and Γ the gamma function), which peaks at ~5 s, convolved with the boxcar function (1 during stimulus presentation, 0 otherwise). After convolution the HRF signal is expected to peak at ~12 s. All pre-processing steps performed on the data were also applied to the HRF signal.

We employed a general linear model (GLM) to quantify the strength between the measured responses to each stimulus condition and the HRF. This model assumes that auditory (*β_a_*) and visual (*β_v_*) inputs independently elicit a hemoglobin concentration change. An extra, third component (*β_av_*) is added in this model, which represents the type and amount of multisensory integration during the presentation of audiovisual stimuli:

(2) $$y(t)=X_{A}(t)\beta_{a}+X_{V}(t)\beta_{\nu }+X_{AV}(t)\beta_{av}+\varepsilon (t)+C$$

with fNIRS data y(t), the explanatory variables X(t), constant regression coefficients β, offset *C* and Gaussian noise ε(*t*).

For each GLM fit, we determined the goodness of fit (*R*^2^-value, and the corresponding *F* and *p*-values). We took the significance of every regression coefficient as a measure of activation compared to baseline, by determining the corresponding *t*- and one-sided *p*-value (larger than 0 for HbO_2_ and smaller than 0 for HbR).

#### Comparisons

To determine whether the beta values differed from a distribution with median 0, the Wilcoxon signed-rank test was applied per cohort. Also, we determined the slope between regression coefficients by determining the optimal fit through simple linear regression. The Wilcoxon Rank Sum test differences in regression coefficients between cohorts were determined.

Significance was assessed at the 0.05 alpha level.

## Results

### Functional NIRS—Representative Single Subject Data

**Figure 5:**
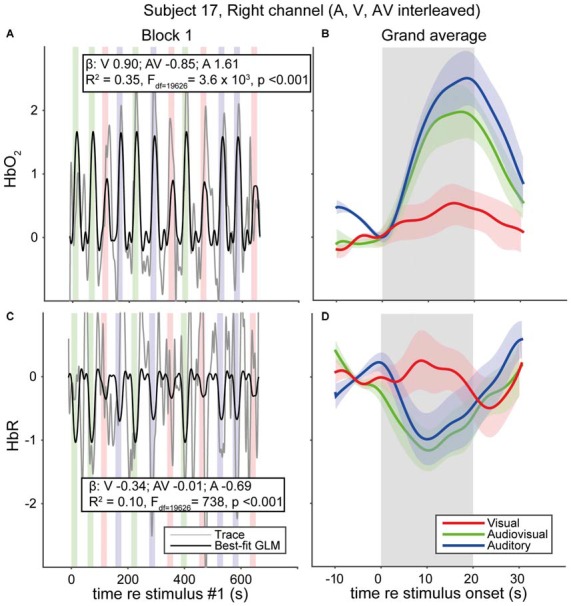
**fNIRS data of a representative normal-hearing subject. (A)** HbO_2_ and **(C)** HbR traces in a single block of a single normal-hearing subject. Average of the 12 **(B)** HbO_2_ and **(D)** HbR responses measured for A, V and AV stimuli. Colors denote stimulus modality; auditory (blue), visual (red) and audiovisual (green). Rectangular patches denote stimulus activation. The best-fit (predicted) canonical hemodynamic response is shown in **(A,C)** as a black line. Insets in **(A,C)** provide the beta values for individual modalities and the goodness of fit. Shaded areas depict standard error of the mean over trials.

We measured fNIRS activity over the temporal cortex of 33 normal-hearing subjects and 5 CI users while they were watching and/or listening to auditory, visual and audiovisual speech stimuli (Figure 5). Individual traces of HbO_2_ and HbR signals for a single representative normal-hearing subject (NH17) generally increase and decrease, respectively, during the stimulus epochs (Figures 5A,B). Still, despite the extensive pre-processing (see “Materials and Methods” Section), signal drift, typical for fNIRS measurements (Sevy et al., 2010), occurs also during the silent dark periods. To deal with this stimulus-independent noise, we averaged the signals over the 12 trials per stimulus modality (Figures 5C,D). The normalized, average HbO_2_ over the 12 auditory-only (A) stimuli increases from baseline at sound onset reaching its maximum after about 15 s (Figure 5C, blue), which is slightly more (~9%) than the average for the 12 AV stimuli (Figure 5C, green). The visual (V) trial average (Figure 5C, red), while also increasing, only reaches a maximum of ~27% of the A maximum. These increases are mirrored in the HbR decreases, albeit with a slightly lower amplitude (Figure 5D). After stimulus offset, HbO_2_ and HbR return gradually to baseline (within 10 s). Typically, and as exemplified for this subject, the hemodynamic response corresponds well to the actual signals (cf. Figures 1, 1, 5B).

### Hemodynamic Response Shapes to Auditory, Visual and Audiovisual Stimulation for Normal-Hearing Subjects and CI Users

**Figure 6:**
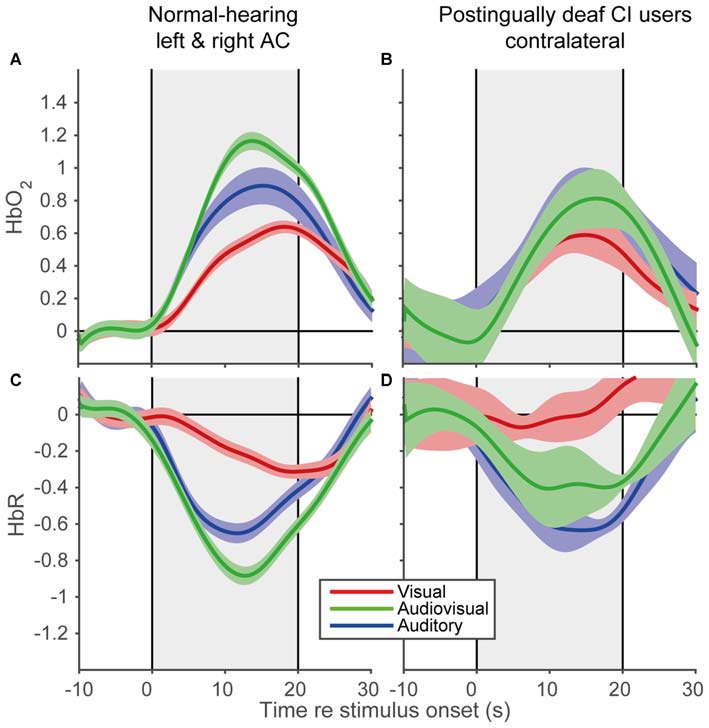
**Grand average hemodynamic response of normal-hearing subjects and CI users.** Grand average responses for HbO_2_ of normal-hearing subjects **(A)** and CI users **(B)**. Grand average responses for HbR of normal-hearing subjects **(C)** and CI users **(D)**. For the normal-hearing subjects 54 channels (21 bilateral, 12 unilateral) and for the CI users five unilateral channels were recorded. Gray rectangular patch denotes stimulus activation. Colors denote: red—visual; blue—auditory; green—audiovisual stimulation. Shaded areas depict standard error of the mean over subjects.

To reveal the shape of the cortical hemodynamic response, we averaged the trial averages over subjects for the A, V, and AV modalities, for the time interval between 10 s before stimulus onset and 10 s after stimulus offset (Figure 6). All modalities demonstrated similar response shapes, albeit with varying amplitudes. The signals changed after stimulus onset (increase for HbO_2_ and decrease for HbR) followed by a recovery back to baseline after stimulus offset. The data of the CI users (Figures 6B,D) exhibited similar trends (Figures 6A,C), although the standard errors were slightly larger (also due to the lower number of subjects in the CI user cohort). Moreover, the observed response resembled the predicted response shape (cf. Figure 1), at least qualitatively. These similarities in response shapes for all cohorts, modalities and prediction indicate that fNIRS can consistently measure temporal cortical responses to auditory and visual stimuli in normal-hearing and cochlear-implanted adults.

### Cortical Hemodynamic Amplitude Changes Reveal Saturation

**Figure 7:**
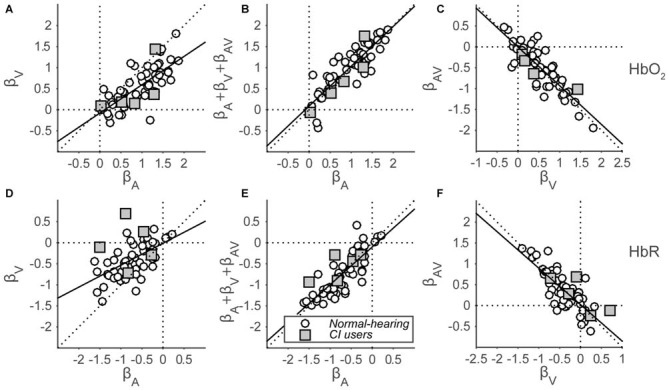
**Beta coefficients of the GLM.** HbO_2_ **(A–C)** and HbR **(D–F)** β-coefficients are shown for all subjects for all stimulus modalities. **(A,D)**: visual vs. auditory regression coefficients. **(B,E)**: summed auditory, visual and AV-interaction (representing the AV response amplitude) vs. auditory regression coefficients. **(C,F)**: audiovisual-interaction vs. visual regression coefficients. Open circles indicate normal-hearing subjects—filled squares indicate CI users. The black line depicts the best-fit regression line.

To quantify the evoked responses, we fitted a GLM (Eqn. 2; see “Materials and Methods” Section) that assumes that auditory and visual inputs independently elicit a hemoglobin amplitude change, also during audiovisual stimulation. An extra, third component is added in this model, which represents the type and amount of multisensory integration during the presentation of audiovisual stimuli. The analysis yields a set of three beta coefficients (Figure 7) representing the modeled amplitude changes for each response component (auditory, visual and audiovisual interaction), for each subject (both cohorts), for both hemispheres (if applicable), and for both HbO_2_ and HbR. In line with the grand average response for the normal-hearing cohort (Figures 6A,C), significant activation was observed for the majority of single HbO_2_ and HbR channels in normal-hearing subjects by auditory and/or visual stimulation (A: 52/54 and 50/54; V: 47/54 and 38/54 regression coefficients were larger/smaller than 0, for HbO_2_ and HbR, respectively). Most channels did not exhibit an additional AV component (AV: 7/54 and 9/54).

In line with the significance of unisensory individual channel activation, the coefficients for both the auditory and visual components reveal a general positive amplitude change for HbO_2_ (Wilcoxon signed-rank test; for auditory coefficients: *p* < 0.001, *z* = 6.8, rank = 1779, and for visual coefficients: *p* < 0.001, *z* = 6.2, rank = 1709) on a group-level, although there is a large intra-coefficient variability, with beta values ranging between −0.3 and 1.9. In contrast, comparisons between coefficients show a systematic trend of visual coefficients being smaller than auditory coefficients (Figure 7A, open circles; Wilcoxon signed-rank test: *p* < 0.001, *z* = 5.8, rank = 1657). A similar, opposite pattern arises for HbR (Figure 7D, Wilcoxon signed-rank test: *p* < 0.001, *z* = −6.6, rank = 11; *p* < 0.001, *z* = −5.3, rank = 182, for A and V, respectively; for comparison between A and V: *p* < 0.001, *z* = −5.1, rank = 203). The auditory data signify that we can reliably obtain auditory responses from temporal cortex with fNIRS, and the slightly weaker visual response data arguably imply that cross-modal, visual activation can arise from the same recording site (see also “Discussion, Multisensory Integration vs. Saturation” Section).

To test for multisensory integration, researchers typically compare the bimodal response to the largest unimodal response (Stein et al., 2009; Figure 1). As the far majority of auditory coefficients are larger than the visual coefficients (HbO_2_: 49 of 54; HbR: 43 of 54), we chose to compare only the auditory response with the bimodal response for all subjects (Figures 7B,E). Note that the bimodal amplitudes are constituted by the sum of the auditory, visual and audiovisual-interaction coefficients (see Methods). These audiovisual amplitudes are highly similar to the auditory coefficients as all points lie close to the unity line, both for HbO_2_ and HbR (Figures 7B,E; Wilcoxon signed-rank test: for HbO_2_ *p* = 0.14, *z* = −1.5, rank = 688, slope: 0.95; for HbR *p* = 0.34, *z* = 0.95, rank = 1011, slope = 0.89).

The audiovisual interaction components are almost exactly inversely related to the visual amplitudes (Figures 7C,F; i.e., data points lie close to *y* = −*x* line, regression slopes: −0.93 and −0.87 for HbO_2_ and HbR, respectively). This might be indicative of a saturation effect as the extra audiovisual interaction effect almost exactly counterbalances any effect a visual component might have (see also “Discussion” Section).

Concentration changes evoked in the five CI users (Figure 7, gray squares) resembled those evoked in the normal-hearing subjects. Specifically, the auditory and visual coefficients for the CI users ranged from 0.02 to 1.4 (Figure 7A) and from −1.5 to 0.7 (Figure 7B) for HbO_2_ and HbR, respectively. Significant activation from baseline for auditory components was observed for 4 out of 5 and 5 out of 5 subjects for HbO_2_ and HbR, respectively. The visual components were significant for 5 out of 5 and 3 out of 5 subjects, respectively. The audiovisual components were significant for 0 out of 5 and 2 out 5 CI users.

## Discussion

### Overview

In this study, we assessed audio-visual activation in temporal cortex with fNIRS. Specifically, we studied cortical activation as present in concentration changes of oxy- and deoxy-hemoglobin of normal-hearing adult subjects and post-lingually deaf unilateral CI users in response to auditory, visual and auditory-visual speech stimuli. Sounds evoked larger concentration changes than visual stimuli (Figures 7A,D). The audiovisual fNIRS signal resembled the purely auditory response (Figures 7B,E) with the visual component being almost exactly inversely related to the audiovisual component (Figures 7C,F). Interestingly, hemodynamic concentration changes evoked in the CI users strongly resembled those of the normal-hearing subjects (Figure 7).

### Feasibility

Since we show robust evoked activity in the temporal cortex for three different sensory conditions in fNIRS data on a group level (Figure 6), fNIRS seems suited to study auditory and visual processing. Furthermore, the responses for the various modalities were consistent when compared against each other within subjects (Figure 7). Nevertheless, a large idiosyncratic variation on single-modality fNIRS responses (Figure 7) may limit the use of this technique on single subject level. The causes for the observed inter-subject variance might be threefold: (1) methodological; (2) analytical; and (3) experimental. First, we will briefly explain and discuss these issues.

A methodological source of inter-subject variability in our data is the placement of the optodes. According to the 10/20 International System, we placed the optodes based on external anatomical landmarks (i.e., nasion and inion; Sevy et al., 2010; Dewey and Hartley, 2015). Alternatively, one could place the optodes based on functional landmarks, by conducting a short functional localizer experiment, such that the location of the maximal response is searched for in a pilot experiment. For example, tone responsiveness could be determined in order to localize basic auditory-responsive regions (Plichta et al., 2011). Another optimization of the current 2-channel optode design would be to use multichannel optode arrays (Pollonini et al., 2014; Chen et al., 2015a; Dewey and Hartley, 2015), so that only the channel(s) with the strongest evoked responses are analyzed (as is current practice, e.g., Chen et al., 2015b), or to determine a clearly localized response (e.g., Kennan et al., 2002; Pollonini et al., 2014; Chen et al., 2015b). In addition, one might consider to determine the individual optode positions in such a multi-optode array from anatomical MRI scans per subject (Barbour et al., 1995; Pogue and Paulsen, 1998; Barnett et al., 2003).

This study reveals that RCS is a very important factor in the analysis (Figure 4). Typically, this is not performed (Scarpa et al., 2013), although it is considered essential in removing systemic noise (Scholkmann et al., 2014). Without RCS, no effect in any of the sensory modalities would be observed in the current data (not shown here, but see Figures 4D,E). A refinement of the current procedure would be to systematically change the inter-optode distances in order to optimally record purely systemic noise (in the reference channel) and the largest evoked hemodynamic signal (in the deep channel). The use of a multichannel optode array with varying optode distances might be ideally suited to disentangle the systemic noise from the evoked signal.

Finally, the experimental paradigm might in itself explain the variability. In this case, it might turn out that idiosyncratic variability is real, and that the amount of neural or hemodynamic activity varies on an individual basis. Variation might then be reduced if the evoked response is maximized for all subjects by specifically tailored experimental paradigms. For example, in the current paradigm, subjects were passively exposed to the stimuli, while active listening typically results in increased cortical activity in humans (Grady et al., 1997; Vannest et al., 2009; Turner et al., 2013) and non-human primates (Wang et al., 2005; Massoudi et al., 2013, 2014; Osmanski and Wang, 2015). Furthermore, one might refine the stimuli in order to elicit optimal responses from the brain area under consideration. Here, we used speech stimuli, although primary auditory and belt areas might be more responsive to basic acoustic stimuli, such as amplitude-modulated Gaussian white noises, or dynamic spectral-temporal ripples. Yet, higher (belt) auditory cortical regions might respond better to more natural stimuli.

### Multisensory Integration vs. Saturation

Our data is in line with cross-sensory influences on neuronal activity, as a clear response was evoked by visual trials over an auditory-responsive, temporal cortical area (Figures 6, 7). This is in line with earlier studies that show a cross-sensory influence on neuronal activity at early cortical areas, which have been traditionally held as unisensory (Ghazanfar et al., 2002; Foxe and Schroeder, 2005; Schroeder and Foxe, 2005; Kayser et al., 2007, 2010; Koelewijn et al., 2010). However, we cannot exclude the possibility that recordings may have partially been taken from higher auditory supplementary areas, as fNIRS records signals arising from a large (1–2 cm) diffuse area (Boas et al., 2004). As such visual-evoked signals might potentially originate from areas in the superior temporal gyrus that encode for face recognition, lip-reading, or other higher-cognitive functions (Sams et al., 1991; Calvert et al., 1997; MacSweeney et al., 2000). The data support the idea that we recorded from predominantly auditory areas, as sounds almost invariantly elicited the largest responses, and the visual activation was nearly completely nulled during audiovisual stimulation (i.e., audiovisual activation was not significantly different from the inverse of visual-only activation, Figures 7C,F).

On a group level, the AV responses hint at auditory dominance (Figure 7), because the visual response, as presented in isolation, does not appear in the AV response. Two distinct mechanisms might underlie this phenomenon. First, a true multisensory integrative effect could have been present (Stein et al., 2009; Figure 1), in which the visual component is effectively counterbalanced by an inhibitory audiovisual integration effect (Figures 7C,F). Alternatively, the hemoglobin response might have reached saturation by the supra-threshold, highly intelligible auditory stimulus. Then, adding a visual stimulus will not lead to a stronger response. It seems unlikely that the nearly exact inverse relationship between the audiovisual and visual components in the audiovisual regression model (Figures 7C,F) would be explained by multisensory integration, as it is precisely expected from a saturation effect. To better dissociate these different interpretations, auditory and visual stimuli should be presented in regimes that prevent neural saturation, and/or better characterize the visual response.

**Figure 8:**
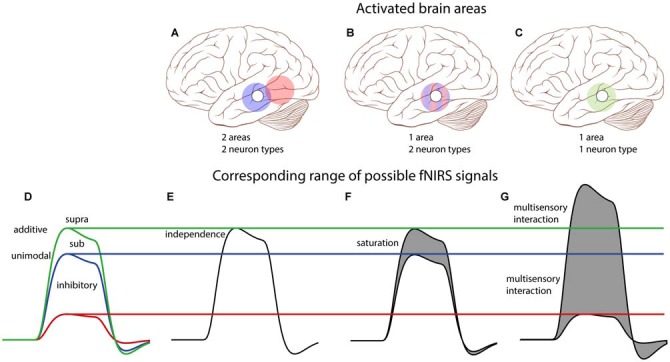
**Conceptual schematic effects of potential audiovisual interactions on single-channel fNIRS signals.** Top: We consider all three possible scenarios: **(A)** two spatially separated areas are each activated by either auditory or visual inputs; **(B)** neurons are either auditory or visually responsive, but are interspersed within one area; **(C)** one area contains bimodal neurons that respond to both auditory and visual stimulation. The open circle on the brain schematically depicts the location of the single T7/8 fNIRS channel, and colored circles depict the activation patterns of indicated areas: blue—auditory, red—visual, green—auditory and visual. Bottom: **(D)** Description of potential integrative effects (see also Figure 1). **(E)** For two independent areas of unisensory neurons (cf. **A**), the audiovisual fNIRS signal (black) can only be the sum of the auditory and visual fNIRS signals (and thus equals the additive model—green in **D**). **(F)** For a mix of unisensory neurons in one area (cf. **B**), both neuron populations will be similarly active for their unisensory-preferred stimulus as for the audiovisual stimulus. The fNIRS signal then equals the additive model, or less (gray area) if saturation of the BOLD response occurs (sub-additive model, between blue and green in **D**). **(G)** For an area with multisensory neurons (cf. **C**), fNIRS signals could yield any response type. Note that only a multisensory-area can generate multisensory interactions like super-additivity (above green), or inhibition (below blue). Parts of this image have been taken from https://commons.wikimedia.org/wiki/File:Skull_and_brain_normal_human.svg. Patrick J. Lynch; C. Carl Jaffe; Yale University Center for Advanced Instructional Media; under Creative Commons Attribution 2.5 License 2006.

Note that with a one-channel setup it is impossible to decide whether the auditory and visual activations originated from the same area, or from spatially separated areas, when the AV response would equal the sum of the A and V responses. However, if the AV response deviates from the purely additive prediction several possibilities may be dissociated, as explained in Figure 8. Importantly, activation of two distinct, independent brain areas (Figures 8A,E) does not predict the saturation that is observed in our data. The sub-additive AV response observed in our resulst (a peak activation between the blue and green lines in Figure 8) allows for two possible scenarios: (i) the signals could have originated from true multisensory neurons (Figures 8C,G), or (ii) from two distinct subpopulations of unisensory-responsive neurons within the recorded area (Figures 8B,F). Note, however, that whenever the peak activation exceeds the additive response (green line), or falls below the strongest unimodal response (blue line), it will be a signature for true multisensory neural integration.

### Post-Lingually Deaf CI Users

The brain can reorganize after sensory deprivation, such as caused by deafness (Rauschecker, 1995; Lee et al., 2001). The question is whether cross-modal reorganization after deafening might introduce stronger visual effects over auditory cortex in post-lingual deaf subjects. This is not the case in our limited group of CI users (Figures 7A,D), as visual activation was lower than auditory evoked activity.

Our cohort of post-lingual deaf CI users did not differ from the normal-hearing cohort with respect to cortical activation for audio-visual stimuli (Figure 7, gray squares). This is seemingly in contrast to the principle of inverse effectiveness, which suggests that people with sensory impairments might benefit from multisensory integration. Specifically, a larger multisensory enhancement compared to the purely auditory response would be predicted because of the hearing-impairment of the CI users (and thus the weaker auditory percepts). This is not observed (Figures 7B,E), indicating that either the stimuli were still supra-threshold for these subjects, or that saturation still dominated the audiovisual responses. Both possibilities imply a paradigm that aims at near-threshold stimulation in order to study this principle. Moreover, a larger cohort of CI users is desired when the issues of supra-threshold stimuli and response saturation have been overcome.

## Conclusion

We found increased activation to auditory, visual and audiovisual stimulation in temporal cortex of normal-hearing subjects and post-lingually deaf CI users using fNIRS. Our findings demonstrate the potential of fNIRS for studying the neural mechanisms of audiovisual integration, both in normal-hearing subjects and in hearing-impaired subjects following cochlear implantation.

## Author Contributions

Conceived and designed the experiments: AFMS, EAMM, LVS, HYH, MMvW. Performed the experiments: LPHvdR, HYH, LVS. Analyzed the data: LPHvdR, MMvW. Wrote the paper: LPHvdR, MMvW, AJvO, AFMS, EAMM.

## Funding

This work was supported by Cochlear Benelux NV (LR, MW, LS), the Radboud University Nijmegen (AJvO), and the RadboudUMC (EAMM, AFMS). The funders had no role in study design, data collection and analysis, decision to publish, or preparation of the manuscript.

## Conflict of Interest Statement

The authors declare that the research was conducted in the absence of any commercial or financial relationships that could be construed as a potential conflict of interest.

## Footnotes

^1^http://www.wma.net/en/30publications/10policies/b3/

## References

[B1] Abdelnour A. F. Huppert T. (2009). Real-time imaging of human brain function by near-infrared spectroscopy using an adaptive general linear model. Neuroimage 46, 133–143. 10.1016/j.neuroimage.2009.01.033 19457389 PMC2758631

[B2] Agterberg M. J. H. Snik A. F. M. M. Hol M. K. S. van Esch T. E. M. M. Cremers C. W. R. J. R. J. Van Wanrooij M. M.. (2011). Improved horizontal directional hearing in bone conduction device users with acquired unilateral conductive hearing loss. J. Assoc. Res. Otolaryngol. 12, 1–11. 10.1007/s10162-010-0235-2 20838845 PMC3015026

[B3] Barbour R. L. Graber H. L. Jenghwa Chang Barbour S.-L. S. Koo P. C. Aronson R. (1995). MRI-guided optical tomography: prospects and computation for a new imaging method. IEEE Comput. Sci. Eng. 2, 63–77. 10.1109/99.476370

[B4] Barnett A. H. Culver J. P. Sorensen A. G. Dale A. Boas D. A. (2003). Robust inference of baseline optical properties of the human head with three-dimensional segmentation from magnetic resonance imaging. Appl. Opt. 42, 3095–3108. 10.1364/ao.42.003095 12790461

[B5] Beauchamp M. S. (2005). See me, hear me, touch me: multisensory integration in lateral occipital-temporal cortex. Curr. Opin. Neurobiol. 15, 145–153. 10.1016/j.conb.2005.03.011 15831395

[B6] Boas D. A. Dale A. M. Franceschini M. A. (2004). Diffuse optical imaging of brain activation: approaches to optimizing image sensitivity, resolution and accuracy. in. Neuroimage 23, S275–S288. 10.1016/j.neuroimage.2004.07.011 15501097

[B7] Brainard D. H. (1997). The psychophysics toolbox. Spat. Vis. 10, 433–436. 10.1163/156856897x00357 9176952

[B8] Bremen P. van Wanrooij M. M. van Opstal A. J. (2010). Pinna cues determine orienting response modes to synchronous sounds in elevation. J. Neurosci. 30, 194–204. 10.1523/JNEUROSCI.2982-09.2010 20053901 PMC6632510

[B9] Brigadoi S. Cooper R. J. (2015). How short is short? Optimum source-detector distance for short-separation channels in functional near-infrared spectroscopy. Neurophotonics 2:025005. 10.1117/1.NPh.2.2.025005 26158009 PMC4478880

[B10] Calvert G. A. Bullmore E. T. Brammer M. J. Campbell R. Williams S. C. McGuire P. K.. (1997). Activation of auditory cortex during silent lipreading. Science 276, 593–596. 10.1126/science.276.5312.593 9110978

[B11] Calvert G. A. Spence C. Stein B. E. (eds) (2004). The Handbook of Multisensory Processing, Cambridge, MA: The MIT Press.

[B12] Chen L.-C. Sandmann P. Thorne J. D. Bleichner M. G. Debener S. (2015a). Cross-modal functional reorganization of visual and auditory cortex in adult cochlear implant users identified with fNIRS. Neural Plasticity 2016:4382656. 10.1155/2016/4382656 PMC4706950 26819766

[B13] Chen L.-C. Sandmann P. Thorne J. D. Herrmann C. S. Debener S. (2015b). Association of concurrent fNIRS and EEG signatures in response to auditory and visual stimuli. Brain Topogr. 28, 710–725. 10.1007/s10548-015-0424-8 25589030

[B14] Cope M. Delpy D. T. (1988). System for long-term measurement of cerebral blood and tissue oxygenation on newborn infants by near infra-red transillumination. Med. Biol. Eng. Comput. 26, 289–294. 10.1007/bf02447083 2855531

[B15] Corneil B. D. Van Wanrooij M. Munoz D. P. Van Opstal A. J. (2002). Auditory-visual interactions subserving goal-directed saccades in a complex scene. J. Neurophysiol. 88, 438–454. 10.1152/jn.00699.2001 12091566

[B16] Cui X. Bray S. Bryant D. M. Glover G. H. Reiss A. L. (2011). A quantitative comparison of NIRS and fMRI across multiple cognitive tasks. Neuroimage 54, 2808–2821. 10.1016/j.neuroimage.2010.10.069 21047559 PMC3021967

[B17] Dewey R. S. Hartley D. E. H. (2015). Cortical cross-modal plasticity following deafness measured using functional near-infrared spectroscopy. Hear. Res. 325, 55–63. 10.1016/j.heares.2015.03.007 25819496

[B18] Fekete T. Rubin D. Carlson J. M. Mujica-Parodi L. R. (2011). The NIRS analysis package: noise reduction and statistical inference. PLoS One 6:e24322. 10.1371/journal.pone.0024322 21912687 PMC3166314

[B19] Foxe J. J. Schroeder C. E. (2005). The case for feedforward multisensory convergence during early cortical processing. Neuroreport 16, 419–423. 10.1097/00001756-200504040-00001 15770144

[B20] Fukui Y. Ajichi Y. Okada E. (2003). Monte Carlo prediction of near-infrared light propagation in realistic adult and neonatal head models. Appl. Opt. 42, 2881–2887. 10.1364/ao.42.002881 12790436

[B21] Ghazanfar A. A. Neuhoff J. G. Logothetis N. K. (2002). Auditory looming perception in rhesus monkeys. Proc. Natl. Acad. Sci. U S A 99, 15755–15757. 10.1073/pnas.242469699 12429855 PMC137788

[B22] Gilley P. M. Sharma A. Dorman M. Finley C. C. Panch A. S. Martin K. (2006). Minimization of cochlear implant stimulus artifact in cortical auditory evoked potentials. Clin. Neurophysiol. 117, 1772–1782. 10.1016/j.clinph.2006.04.018 16807102

[B23] Grady C. L. Van Meter J. W. Maisog J. M. Pietrini P. Krasuski J. Rauschecker J. P. (1997). Attention-related modulation of activity in primary and secondary auditory cortex. Neuroreport 8, 2511–2516. 10.1097/00001756-199707280-00019 9261818

[B24] Haeussinger F. B. Heinzel S. Hahn T. Schecklmann M. Ehlis A.-C. Fallgatter A. J. (2011). Simulation of near-infrared light absorption considering individual head and prefrontal cortex anatomy: implications for optical neuroimaging. PLoS One 6:e26377. 10.1371/journal.pone.0026377 22039475 PMC3200329

[B25] Hall D. A. Haggard M. P. Akeroyd M. A. Summerfield A. Q. Palmer A. R. Elliott M. R.. (2000). Modulation and task effects in auditory processing measured using fMRI. Hum. Brain Mapp. 10, 107–119. 10.1002/1097-0193(200007)10:3<107::aid-hbm20>3.3.co;2-# 10912590 PMC6871907

[B26] Helfer K. S. (1997). Auditory and auditory-visual perception of clear and conversational speech. J. Speech Lang. Hear. Res. 40, 432–443. 10.1044/jslhr.4002.432 9130211

[B27] Henson R. Friston K. (2007). “Convolution models for fMRI”, in Statistical Parametric Mapping: The Analysis of Functional Brain Images (Amsterdam: Elsevier), 178–192.

[B28] Herwig U. Satrapi P. Schönfeldt-Lecuona C. (2003). Using the international 10–20 EEG system for positioning of transcranial magnetic stimulation. Brain Topogr. 16, 95–99. 10.1023/b:brat.0000006333.93597.9d 14977202

[B29] Huppert T. J. Allen M. S. Diamond S. G. Boas D. A. (2009a). Estimating cerebral oxygen metabolism from fMRI with a dynamic multicompartment Windkessel model. Hum. Brain Mapp. 30, 1548–1567. 10.1002/hbm.20628 18649348 PMC2670946

[B30] Huppert T. J. Diamond S. G. Franceschini M. A. Boas D. A. (2009b). HomER: a review of time-series analysis methods for near-infrared spectroscopy of the brain. Appl. Opt. 48, D280–D298. 10.1364/ao.48.00d280 19340120 PMC2761652

[B31] Huppert T. J. Hoge R. D. Dale A. M. Franceschini M. A. Boas D. A. (2006). Quantitative spatial comparison of diffuse optical imaging with blood oxygen level-dependent and arterial spin labeling-based functional magnetic resonance imaging. J. Biomed. Opt. 11:064018. 10.1117/1.2400910 17212541 PMC2670188

[B32] Jasper H. H. (1958). Report of the committee on methods of clinical examination in electroencephalography. Electroencephalogr. Clin. Neurophysiol. 10, 370–375. 10.1016/0013-4694(58)90053-1

[B33] Jöbsis F. F. (1977). Noninvasive, infrared monitoring of cerebral and myocardial oxygen sufficiency and circulatory parameters. Science 198, 1264–1267. 10.1126/science.929199 929199

[B34] Johnsrude I. S. Giraud A. L. Frackowiak R. S. J. (2002). Functional imaging of the auditory system: the use of positron emission tomography. Audiol. Neurootol. 7, 251–276. 10.1159/000064446 12232496

[B35] Julien C. (2006). The enigma of Mayer waves: facts and models. Cardiovasc. Res. 70, 12–21. 10.1016/j.cardiores.2005.11.008 16360130

[B36] Kayser C. Logothetis N. K. Panzeri S. (2010). Visual enhancement of the information representation in auditory cortex. Curr. Biol. 20, 19–24. 10.1016/j.cub.2009.10.068 20036538

[B37] Kayser C. Petkov C. I. Augath M. Logothetis N. K. (2007). Functional imaging reveals visual modulation of specific fields in auditory cortex. J. Neurosci. 27, 1824–1835. 10.1523/jneurosci.4737-06.2007 17314280 PMC6673538

[B38] Kennan R. P. Horovitz S. G. Maki A. Yamashita Y. Koizumi H. Gore J. C. (2002). Simultaneous recording of event-related auditory oddball response using transcranial near infrared optical topography and surface EEG. Neuroimage 16, 587–592. 10.1006/nimg.2002.1060 12169245

[B39] Kleiner M. Brainard D. Pelli D. (2007). What’s new in Psychtoolbox-3. Perception 36 (ECVP Abstr. Suppl.), 1–235. 10.1177/03010066070360S101

[B40] Kocsis L. Herman P. Eke A. (2006). The modified Beer-Lambert law revisited. Phys. Med. Biol. 51, N91–N98. 10.1088/0031-9155/51/5/n02 16481677

[B41] Koelewijn T. Bronkhorst A. Theeuwes J. (2010). Attention and the multiple stages of multisensory integration: a review of audiovisual studies. Acta Psychol. (Amst) 134, 372–384. 10.1016/j.actpsy.2010.03.010 20427031

[B42] Laurienti P. J. Perrault T. J. Stanford T. R. Wallace M. T. Stein B. E. (2005). On the use of superadditivity as a metric for characterizing multisensory integration in functional neuroimaging studies. Exp. Brain Res. 166, 289–297. 10.1007/s00221-005-2370-2 15988597

[B43] Lee D. S. Lee J. S. Oh S. H. Kim S. K. Kim J. W. Chung J. K.. (2001). Cross-modal plasticity and cochlear implants. Nature 409, 149–150. 10.1038/35051653 11196628

[B44] Lindquist M. A. Meng Loh J. Atlas L. Y. Wager T. D. (2009). Modeling the hemodynamic response function in fMRI: efficiency, bias and mis-modeling. Neuroimage 45, S187–S198. 10.1016/j.neuroimage.2008.10.065 19084070 PMC3318970

[B45] MacLeod A. Summerfield Q. (1990). A procedure for measuring auditory and audio-visual speech-reception thresholds for sentences in noise: rationale, evaluation and recommendations for use. Br. J. Audiol. 24, 29–43. 10.3109/03005369009077840 2317599

[B46] MacSweeney M. Amaro E. Calvert G. A. Campbell R. David A. S. McGuire P.. (2000). Silent speechreading in the absence of scanner noise: an event-related fMRI study. Neuroreport 11, 1729–1733. 10.1097/00001756-200006050-00026 10852233

[B47] Malinen S. Hlushchuk Y. Hari R. (2007). Towards natural stimulation in fMRI—Issues of data analysis. Neuroimage 35, 131–139. 10.1016/j.neuroimage.2006.11.015 17208459

[B48] Massoudi R. Van Wanrooij M. M. Van Wetter S. M. C. I. Versnel H. Van Opstal A. J. (2013). Stable bottom-up processing during dynamic top-down modulations in monkey auditory cortex. Eur. J. Neurosci. 37, 1830–1842. 10.1111/ejn.12180 23510187

[B49] Massoudi R. Van Wanrooij M. M. Van Wetter S. M. C. I. Versnel H. Van Opstal A. J. (2014). Task-related preparatory modulations multiply with acoustic processing in monkey auditory cortex. Eur. J. Neurosci. 39, 1538–1550. 10.1111/ejn.12532 24649904

[B50] Niedermeyer E. Lopes da Silva F. (2005). Electroencephalography: Basic Principles Clinical Applications and Related Fields. Lippincott Williams and Wilkins, eds Schomer D. L. da Silva F. L. (Philadelphia, PA: Lippincott Williams and Wilkins), 1309.

[B51] Osmanski M. S. Wang X. (2015). Behavioral dependence of auditory cortical responses. Brain Topogr. 28, 365–378. 10.1007/s10548-015-0428-4 25690831 PMC4409507

[B52] Pei Y. Wang Z. Barbour R. L. (2007). “NAVI-SciPort solution: a problem solving environment (PSE) for nirs data analysis,” in Poster at Human Brain Mapping, Chicago, IL.

[B53] Pelli D. G. (1997). The VideoToolbox software for visual psychophysics: transforming numbers into movies. Spat. Vis. 10, 437–442. 10.1163/156856897x00366 9176953

[B54] Plichta M. M. Gerdes A. B. M. Alpers G. W. Harnisch W. Brill S. Wieser M. J.. (2011). Auditory cortex activation is modulated by emotion: a functional near-infrared spectroscopy (fNIRS) study. Neuroimage 55, 1200–1207. 10.1016/j.neuroimage.2011.01.011 21236348

[B55] Pogue B. W. Paulsen K. D. (1998). High-resolution near-infrared tomographic imaging simulations of the rat cranium by use of a priori magnetic resonance imaging structural information. Opt. Lett. 23, 1716–1718. 10.1364/ol.23.001716 18091894

[B56] Pollonini L. Olds C. Abaya H. Bortfeld H. Beauchamp M. S. Oghalai J. S. (2014). Auditory cortex activation to natural speech and simulated cochlear implant speech measured with functional near-infrared spectroscopy. Hear. Res. 309, 84–93. 10.1016/j.heares.2013.11.007 24342740 PMC3939048

[B57] Rauschecker J. P. (1995). Compensatory plasticity and sensory substitution in the cerebral cortex. Trends Neurosci. 18, 36–43. 10.1016/0166-2236(95)93948-w 7535489

[B58] Sams M. Aulanko R. Hamalainen M. Hari R. Lounasmaa O. V. Lu S. T.. (1991). Seeing speech: visual information from lip movements modifies activity in the human auditory cortex. Neurosci. Lett. 127, 141–145. 10.1016/0304-3940(91)90914-f 1881611

[B59] Santosa H. Hong M. J. Hong K.-S. (2014). Lateralization of music processing with noises in the auditory cortex: an fNIRS study. Front. Behav. Neurosci. 8:418. 10.3389/fnbeh.2014.00418 25538583 PMC4260509

[B60] Scarpa F. Brigadoi S. Cutini S. Scatturin P. Zorzi M. Dell’Acqua R.. (2013). A reference-channel based methodology to improve estimation of event-related hemodynamic response from fNIRS measurements. Neuroimage 72, 106–119. 10.1016/j.neuroimage.2013.01.021 23357074

[B61] Scholkmann F. Kleiser S. Metz A. J. Zimmermann R. Mata Pavia J. Wolf U.. (2014). A review on continuous wave functional near-infrared spectroscopy and imaging instrumentation and methodology. Neuroimage 85, 6–27. 10.1016/j.neuroimage.2013.05.004 23684868

[B62] Schroeder C. E. Foxe J. (2005). Multisensory contributions to low-level, “unisensory” processing. Curr. Opin. Neurobiol. 15, 454–458. 10.1016/j.conb.2005.06.008 16019202

[B63] Sevy A. B. G. Bortfeld H. Huppert T. J. Beauchamp M. S. Tonini R. E. Oghalai J. S. (2010). Neuroimaging with near-infrared spectroscopy demonstrates speech-evoked activity in the auditory cortex of deaf children following cochlear implantation. Hear. Res. 270, 39–47. 10.1016/j.heares.2010.09.010 20888894 PMC2997935

[B64] Smith S. M. (2004). Overview of fMRI analysis. Br. J. Radiol. 77, S167–S175. 10.1259/bjr/33553595 15677358

[B66] Stein B. E. (2012). The New Handbook of Multisensory Processes, Cambridge, MA: The MIT Press.

[B65] Stein B. B. E. Meredith M. A. (1993). The Merging of the Senses. Cambridge, MA: The MIT Press.

[B67] Stein B. E. Stanford T. R. Ramachandran R. Perrault T. J. Rowland B. A. (2009). Challenges in quantifying multisensory integration: alternative criteria, models and inverse effectiveness. Exp. Brain Res. 198, 113–126. 10.1007/s00221-009-1880-8 19551377 PMC3056521

[B68] Steinbrink J. Villringer A. Kempf F. Haux D. Boden S. Obrig H. (2006). Illuminating the BOLD signal: combined fMRI-fNIRS studies. Magn. Reson. Imaging 24, 495–505. 10.1016/j.mri.2005.12.034 16677956

[B69] Strangman G. E. Zhang Q. Li Z. (2014). Scalp and skull influence on near infrared photon propagation in the Colin27 brain template. Neuroimage 85, 136–149. 10.1016/j.neuroimage.2013.04.090 23660029

[B70] Themelis G. Selb J. Thaker S. Stott J. J. Custo A. Boas D. A. (2004). “Depth of arterial oscillation resolved with NIRS time and frequency domain,” in Biomedical Topical Meeting (Washington, DC: OSA), WF2.

[B71] Turner B. M. Forstmann B. U. Wagenmakers E.-J. Brown S. D. Sederberg P. B. Steyvers M. (2013). A Bayesian framework for simultaneously modeling neural and behavioral data. Neuroimage 72, 193–206. 10.1016/j.neuroimage.2013.01.048 23370060 PMC4140412

[B72] Van Barneveld D. C. P. B. M. Van Wanrooij M. M. (2013). The influence of static eye and head position on the ventriloquist effect. Eur. J. Neurosci. 37, 1501–1510. 10.1111/ejn.12176 23463919

[B73] Vannest J. J. Karunanayaka P. R. Altaye M. Schmithorst V. J. Plante E. M. Eaton K. J.. (2009). Comparison of fMRI data from passive listening and active-response story processing tasks in children. J. Magn. Reson. Imaging 29, 971–976. 10.1002/jmri.21694 19306445 PMC2763568

[B74] Wang X. Lu T. Snider R. K. Liang L. (2005). Sustained firing in auditory cortex evoked by preferred stimuli. Nature 435, 341–346. 10.1038/nature03565 15902257

[B75] Yamada T. Umeyama S. Matsuda K. (2012). Separation of fNIRS signals into functional and systemic components based on differences in hemodynamic modalities. PLoS One 7:e50271. 10.1371/journal.pone.0050271 23185590 PMC3501470

